# Vaccination is an integral strategy to combat antimicrobial resistance

**DOI:** 10.1371/journal.ppat.1011379

**Published:** 2023-06-15

**Authors:** Liam P. Mullins, Emily Mason, Kaitlin Winter, Manish Sadarangani

**Affiliations:** 1 Experimental Medicine Program, Department of Medicine, University of British Columbia, Vancouver, Canada; 2 Vaccine Evaluation Center, BC Children’s Hospital Research Institute, Vancouver, Canada; 3 Department of Pediatrics, University of British Columbia, Vancouver, Canada; Tufts Univ School of Medicine, UNITED STATES

## Introduction

**Fig 1:**
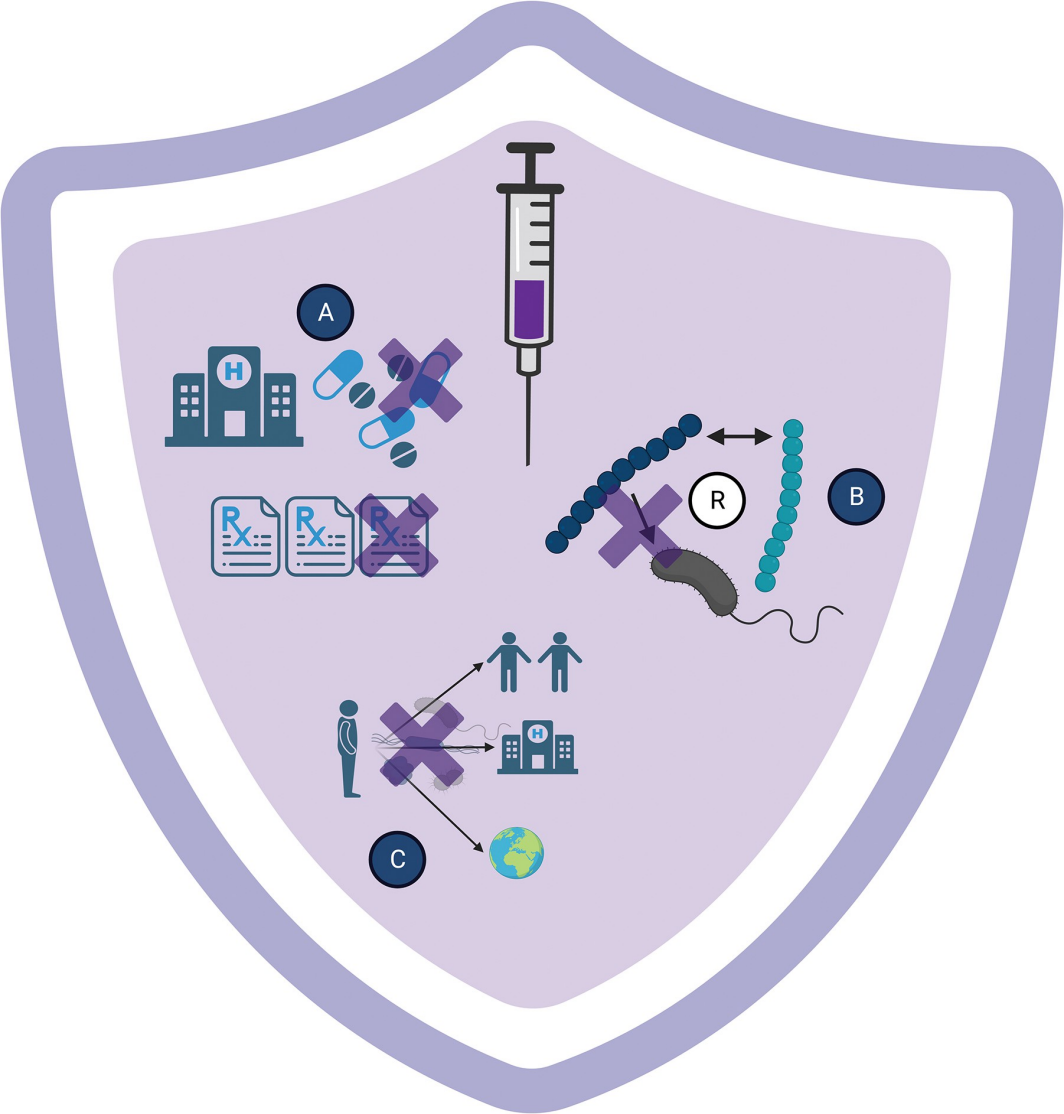
Vaccination as an ideal strategy to reduce antimicrobial resistance. Presently, antibiotics are often overprescribed and overutilized, which leads to acquisition of resistance, and increased spread of resistant bacteria in the community, healthcare settings, and globally. Vaccines targeting resistant bacteria would decrease (A) infections and thus antibiotic prescriptions and use, (B) lower the spread of resistance genes, and (C) reduce transmission in the community, healthcare, and ultimately, globally. Created with BioRender.com.

Antimicrobial-resistant bacterial infections pose a significant challenge to health worldwide. In 2019, there were an estimated 1.95 million deaths and 47.9 million lost disability-adjusted life-years attributable to antimicrobial resistance (AMR) [1]. Over the last 30 years, there has been a stall in the development of new antibiotics while incidence rates of AMR climb [2]. The global AMR crisis is on-track to cause approximately 10 million deaths annually by 2050 [1]. Several pathogens contribute to this, including *Escherichia coli*, *Klebsiella pneumoniae*, *Streptococcus pneumoniae*, *Haemophilus influenzae*, and *Mycobacterium tuberculosis* [1,3]. Targeted interventions to combat AMR, such as vaccines, are essential in conjunction with the continued pursuit of antibiotic discovery and engagement with equitable antibiotic stewardship policies. Many bacterial vaccines are already included in publicly funded vaccination programs and developing technologies in vaccine platforms has the potential to address AMR equitably and effectively [4]. Vaccines have the potential to reduce antibiotic usage at the population level, reduce the spread of bacterial resistance determinants, and decrease transmission of resistant bacteria (Fig 1).

## 1. Why is vaccination an optimal strategy to combat antimicrobial resistance?

Antibiotics are not being developed at the speed necessary to keep up with the evolution of bacterial AMR, with only 13 new drugs in Phase III clinical trials as of a March 2021 report [5]. More than 20 novel antibiotic classes were introduced into clinical practice prior to the 1960s with few new classes marketed since [6]. With this dwindling antibiotic armamentarium, some have suggested that we are in a “post-antibiotic era” [5]. Strategies are needed to preserve these drugs for when they are truly needed. Non-vaccine approaches include implementation of infection control and antibiotic stewardship policies for the optimal use of existing antibiotics. Antibiotics may be co-formulated with compounds such as β-lactamase inhibitors, which enhance antibiotic activity by preventing hydrolysis via bacterial enzymes, or streptazolin which enhances macrophage-mediated killing, potentiating antibiotic action by targeting host cells [7]. In addition, there is a mounting interest in the use of phage therapy as an alternative treatment to antibiotics [8].

Vaccination is an integral strategy to prevent AMR, and reduces infections caused by both antibiotic-susceptible and -resistant bacteria, thereby reducing the overall usage of antibiotics. The success of vaccines in reducing disease burden over the last century is clear; vaccines against *Corynebacterium diphtheriae*, *Neisseria meningitidis*, *Haemophilus influenzae* type b (Hib), and *Bordetella pertussis* resulted in notable decreases in cases [9]. The introduction of pneumococcal vaccines has resulted in a decrease in both invasive disease and a decline in nasopharyngeal carriage of *S*. *pneumoniae* (including antibiotic-resistant isolates), demonstrating vaccination combating AMR in action [10]. Beyond human health, vaccines have been an extremely effective intervention for animal health and have been demonstrated to reduce antimicrobial consumption across many species, including fish, pigs, and poultry [11]. Vaccines against resistant bacterial species also tackle AMR by reducing circulation of drug-susceptible strains that may acquire resistance through horizontal gene transfer [12]. This alleviation of resistance spread sustains the efficacy of antibiotics in combination with antimicrobial stewardship policy. Using one of the most effective medical interventions of the last century (vaccines) to rescue another (antibiotics) is a worthwhile endeavor to allow future generations to benefit from both.

## 2. Who would vaccines against antimicrobial-resistant bacteria benefit most?

As the global burden associated with AMR grows, interventions must be directed towards populations at highest risk of infection and death, including those in low- and middle-income countries (LMICs), immunocompromised and other individuals with comorbidities, and children, who are often excluded from studies investigating novel antimicrobials. Preventing infections is key; in regions, limited access to antibiotics and/or lack of strong antibiotic regulation challenges successful treatment of drug-sensitive infections [13]. In 2019, the estimated all-age mortality rate attributable to AMR was 98.9 deaths per 100,000 in western sub-Saharan Africa, a rate nearly double that of high-income nations [1]. In this region, *K*. *pneumoniae* was responsible for the largest portion of deaths attributable to AMR, at 19.9% of deaths attributed to AMR [1]. Urgent development of vaccines targeting resistant pathogens associated with high morbidity and mortality should be prioritized for development [14].

Individuals with comorbidities have higher risk of infection not only as a direct result of their disease and treatments of it, but through increased hospital exposure, frequent invasive procedures, and as a result, increased antimicrobial usage [15]. Diabetes mellitus is a key example of this, with a notable association with increased susceptibility to *E*. *coli* infection with a larger bacterial burden [16]. People with diabetes may have impaired granulocyte function and high glucose levels impact epithelial barrier functions, predisposing these individuals to increased *E*. *coli* burden in the bladder [16]. Drug-resistant *E*. *coli* contribute to the most deaths of any resistant bacterium worldwide and the increased risk of *E*. *coli* infection in people with diabetes is just one example of a scenario where vaccines against resistant bacteria should be prioritized for specific groups.

Special formulations of antibiotic drugs intended for pediatric use are often developed after the antibiotic has been marketed in adults, leaving children behind in accessing effective antibiotics. Pediatric antimicrobial development includes several hurdles, with different dosages and/or formulations needed that lead to technological and regulatory challenges [17]. Yet, it has been noted that children are some of the highest antibiotic users in any age group, outside of the elderly [17]. Vaccination against bacterial AMR is particularly useful for those groups who struggle to access antimicrobials or face higher rates of bacterial infections due to both geographical and personal health factors.

## 3. How do antimicrobial-resistant bacteria challenge vaccine development?

While vaccination is an optimal strategy to combat AMR in many ways, bacteria can challenge vaccine development through their high genetic diversity, a range of potential antigens and the ability to cause a variety of infections with differing outcomes depending on the status of the host. The genetic heterogeneity of many bacterial pathogens leads to a diversity of structures in protein and polysaccharide antigens, rendering vaccine development difficult and strain cross-protection unlikely [18]. Multicomponent vaccines are commonly used for bacteria to target many antigens to broaden coverage of the vaccine and prevent vaccine resistance due to mutations; however, identifying multiple conserved and immunogenic antigens is challenging. Consider the example of *K*. *pneumoniae*, a major cause of sepsis with increasing rates of AMR [19]. With more than 70 capsular serotypes worldwide, adequate strain coverage with a capsule-based vaccine (as has been used for other bacteria such as *S*. *pneumoniae*) may not be feasible [18]. Bacillus Calmette–Guerin (BCG) has been used as a vaccine against *M*. *tuberculosis* (TB) for nearly a century and is effective in protecting very young children [20]. However, its effectiveness in older children and adults is variable (0% to 80%). This variety in effectiveness may be explained in part by the heterogeneous nature of disease caused by TB, with the optimal immune response to clear infection differing between children and adults. Thus, when vaccines are developed with the goal of combatting AMR both host and pathogen need to be considered. Identification of the target population for the vaccine and an understanding of protective immune responses within that population are vital. In addition, an understanding of which strains have the highest burden, high-quality molecular epidemiology studies, and appropriate antigen selection methods are required.

## 4. Which vaccine platforms have been used for bacterial vaccine development and where might they lead?

Several approaches exist in bacterial vaccines that are currently included in routine immunization schedules, including whole inactivated bacteria, live-attenuated, and subunit vaccines including toxoid and conjugate vaccines. These approaches can include monovalent or multivalent formulations targeting more than 1 antigen. Other platforms have been investigated in recent clinical studies, including the application of nanoparticles, such as outer bacterial membrane vesicles (OMVs), and nucleic acid vaccines that may include DNA-based plasmids or RNA-encoding target antigens [21]. The introduction of mRNA technology to immunization programs with COVID-19 vaccines has the potential to revolutionize the vaccinology field. mRNA vaccines take advantage of host cells for antigen expression making them easily adapted to new pathogens and/or antigens [21]. However, the expression of bacterial proteins in a eukaryotic cell after mRNA vaccination has yet to be investigated in clinical trials. It is likely only a matter of time before this has been addressed, with mRNA-based vaccine candidates targeting Group A or Group B Streptococci demonstrated to be effective at conferring protection in mouse models [22]. Ultimately, the ideal vaccine approach for a specific pathogen must be tailored to the pathology of the specific bacterium.

## 5. Which types of bacterial antigens have been investigated for vaccine development and can they be applied in AMR vaccines?

Current bacterial vaccines are demonstrably effective at reducing disease burden and consequently address AMR via reduction of antibiotic usage. Both pneumococcal and Hib vaccines target the capsular polysaccharides (CPS) of their bacterial species, *S*. *pneumoniae* and *H*. *influenzae*, respectively [3]. The pneumococcal conjugate vaccine is estimated to be 86% to 97% effective against invasive pneumococcal disease, and rates of Hib cases have significantly decreased globally since the introduction of Hib vaccination into the routine childhood immunization programs [3]. Thus, the CPS is one of the most widely targeted and efficacious bacterial antigens in vaccine development. As of 2021, several vaccines for bacterial species with high AMR burden targeting CPS were in clinical trials, such as the 12-valent vaccine against extraintestinal pathogenic *E*. *coli* (ExPEC) [23]. Bioinformatics pipelines allow identification of conserved antigens across heterogeneous populations that may not be selected through traditional vaccinology techniques using whole-genome sequencing of pathogens or convalescent postinfection samples. This approach has been successful for *N*. *meningitidis* and is being used for other vaccines and could help address the high diversity of AMR strains.

## Conclusion

As we look to the future, there are several vaccines in the global vaccine development pipeline that the WHO has identified as having high potential to reduce global AMR [23]. This includes licensed vaccines, some of which have been mentioned above, against *Salmonella enterica* Typhimurium, Hib, and *S*. *pneumoniae*. Vaccines that are currently being tested in Phase III clinical trials include those against extraintestinal pathogenic *E*. *coli*, *Salmonella enterica* ParaTyphi A, and *N*. *gonorrhoeae*. These vaccines target bacteria that have increasing rates of resistance and will reduce both susceptible and resistant infections. Unfortunately, based on the current lack of progress in vaccine development, the WHO has identified several resistant pathogens for which vaccine development is currently thought to have lower feasibility and recommends resources be focused on other tools for the prevention and control of AMR in these bacteria.

AMR is one of the most complex and pressing issues facing modern medicine; vaccines will be a critical part of our strategy to combat rising AMR-associated morbidity and mortality and will be more successful against some pathogens over others. Most vaccines that protect against bacterial infections target common antigens such as the capsular polysaccharide. Moving forward, newly developing technology in vaccine platforms and novel antigen discovery should continue to be explored in preclinical and clinical models. The scientific community must maintain equitable production and distribution as a fundamental element of the vaccine development pipeline. This will ensure that vaccines targeting bacterial AMR will reach the populations who need it most.

## References

[1] Antimicrobial Resistance C. Global burden of bacterial antimicrobial resistance in 2019: a systematic analysis. Lancet. 2022;399(10325):629–655. Epub 20220119. doi: 10.1016/S0140-6736(21)02724-0 ; PubMed Central PMCID: PMC8841637. 35065702 PMC8841637

[2] Plackett B. Why big pharma has abandoned antibiotics. Nature (London). 2020;586(7830):S50–S52. doi: 10.1038/d41586-020-02884-3

[3] Henriques-Normark B, Normark S. Bacterial vaccines and antibiotic resistance. Ups J Med Sci. 2014;119(2):205–208. Epub 20140403. doi: 10.3109/03009734.2014.903324 ; PubMed Central PMCID: PMC4034560. 24694025 PMC4034560

[4] Control BCfD. Vaccines in BC 2023 [cited 2023 January 18]. Available from: http://www.bccdc.ca/health-professionals/clinical-resources/vaccines-in-bc.

[5] Kwon JH, Powderly WG. The post-antibiotic era is here. Science (American Association for the Advancement of Science). 2021;373(6554):471. doi: 10.1126/science.abl5997 34326211

[6] Coates AR, Halls G, Hu Y. Novel classes of antibiotics or more of the same? Br J Pharmacol. 2011;163(1):184–194. doi: 10.1111/j.1476-5381.2011.01250.x ; PubMed Central PMCID: PMC3085877. 21323894 PMC3085877

[7] Wright GD. Antibiotic Adjuvants: Rescuing Antibiotics from Resistance. Trends Microbiol. 2016;24(11):862–871. Epub 20160715. doi: 10.1016/j.tim.2016.06.009 . 27430191

[8] Lin DM, Koskella B, Lin HC. Phage therapy: An alternative to antibiotics in the age of multi-drug resistance. World J Gastrointest Pharmacol Ther. 2017;8(3):162–173. doi: 10.4292/wjgpt.v8.i3.162 ; PubMed Central PMCID: PMC5547374. 28828194 PMC5547374

[9] Pollard AJ, Bijker EM. A guide to vaccinology: from basic principles to new developments. Nat Rev Immunol. 2021;21(2):83–100. doi: 10.1038/s41577-020-00479-7 33353987 PMC7754704

[10] Dagan R. Impact of pneumococcal conjugate vaccine on infections caused by antibiotic-resistant Streptococcus pneumoniae. Clin Microbiol Infect. 2009;15:16–20. doi: 10.1111/j.1469-0691.2009.02726.x 19366365

[11] Hoelzer K, Bielke L, Blake DP, Cox E, Cutting SM, Devriendt B, et al. Vaccines as alternatives to antibiotics for food producing animals. Part 1: challenges and needs. Vet Res. 2018;49(1):64. doi: 10.1186/s13567-018-0560-8 30060757 PMC6066911

[12] Jansen KU, Gruber WC, Simon R, Wassil J, Anderson AS. The impact of human vaccines on bacterial antimicrobial resistance. A review. Environ Chem Lett. 2021;19(6):4031–4062. doi: 10.1007/s10311-021-01274-z 34602924 PMC8479502

[13] Cox JA, Vlieghe E, Mendelson M, Wertheim H, Ndegwa L, Villegas MV, et al. Antibiotic stewardship in low- and middle-income countries: the same but different? Clin Microbiol Infect. 2017;23(11):812–818. Epub 20170714. doi: 10.1016/j.cmi.2017.07.010 . 28712667

[14] Wang LM, Cravo Oliveira Hashiguchi T, Cecchini M. Impact of vaccination on carriage of and infection by antibiotic-resistant bacteria: a systematic review and meta-analysis. Clin Exp. Vaccine Res. 2021;10(2):81–92. Epub 20210531. doi: 10.7774/cevr.2021.10.2.81 ; PubMed Central PMCID: PMC8217572. 34222121 PMC8217572

[15] Lyster H. Antimicrobial stewardship in the immunocompromised patient. Antimicrobial Stewardship. Oxford University Press; 2016.

[16] Mohanty S, Kamolvit W, Scheffschick A, Björklund A, Tovi J, Espinosa A, et al. Diabetes downregulates the antimicrobial peptide psoriasin and increases E. coli burden in the urinary bladder. Nat Commun. 2022;13(1):4983. doi: 10.1038/s41467-022-32636-y 36127330 PMC9489794

[17] Fay MJ, Bryant PA. Antimicrobial stewardship in children: Where to from here? J Paediatr Child Health. 2020;56(10):1504–1507. doi: 10.1111/jpc.15209 33099822

[18] Bekeredjian-Ding I. Challenges for Clinical Development of Vaccines for Prevention of Hospital-Acquired Bacterial Infections. Front Immunol. 2020;11:1755. Epub 20200805. doi: 10.3389/fimmu.2020.01755 ; PubMed Central PMCID: PMC7419648. 32849627 PMC7419648

[19] Assoni L, Girardello R, Converso TR, Darrieux M. Current Stage in the Development of Klebsiella pneumoniae Vaccines. Infect Dis Ther. 2021;10(4):2157–2175. Epub 20210902. doi: 10.1007/s40121-021-00533-4 ; PubMed Central PMCID: PMC8412853. 34476772 PMC8412853

[20] Kumar P. A Perspective on the Success and Failure of BCG. Front Immunol. 2021;12:778028. Epub 20211214. doi: 10.3389/fimmu.2021.778028 ; PubMed Central PMCID: PMC8712472. 34970263 PMC8712472

[21] Kallen KJ, Theß A. A development that may evolve into a revolution in medicine: mRNA as the basis for novel, nucleotide-based vaccines and drugs. Ther Adv Vaccines. 2014;2(1):10–31. doi: 10.1177/2051013613508729 ; PubMed Central PMCID: PMC3991152. 24757523 PMC3991152

[22] Maruggi G, Chiarot E, Giovani C, Buccato S, Bonacci S, Frigimelica E, et al. Immunogenicity and protective efficacy induced by self-amplifying mRNA vaccines encoding bacterial antigens. Vaccine. 2017;35(2):361–368. doi: 10.1016/j.vaccine.2016.11.040 27939014

[23] World Health Organization. Bacterial vaccines in clinical and preclinical development: an overview and analysis. Geneva. 2022.

